# Probiotic-Fermented Mare’s Milk Ameliorates High-Fat Diet-Induced Dyslipidemia Through Coordinated Gut-Liver Metabolic Remodeling

**DOI:** 10.3390/foods15203680

**Published:** 2026-10-16

**Authors:** Rui Zhang, Chao Yin, Naizire Paerhati, Jianbao Ma, Nayilan Zainiding, Sumaierye Batuer, Gulibahaer Kawuli

**Affiliations:** 1 School of Pharmacy, Xinjiang Medical University, Urumqi 830017, China; 18733026972@163.com (R.Z.);; 2 School of Nursing, Xinjiang Medical University, Urumqi 830017, China

**Keywords:** *Lactiplantibacillus plantarum*, fermented mare’s milk, hyperlipidemia, gut microbiota, gut–liver axis, *PPARα* signaling

## Abstract

Dyslipidemia is closely associated with disturbances in hepatic lipid metabolism and gut-liver metabolic homeostasis. This study evaluated the effects of fermented mare’s milk (FMM), produced using an equal-ratio consortium of four cholesterol-lowering Lactiplantibacillus plantarum Ni2 strains, on high-fat diet (HFD)-induced dyslipidemia and characterized the associated gut–liver metabolic responses. Sixty Sprague–Dawley rats were assigned to control, HFD model, simvastatin, and three FMM groups (6, 8, and 10 mL/kg) for 7 weeks. Lipid profiles and liver histology were assessed, and the high-dose FMM group was further analyzed using 16S rRNA sequencing, fecal and hepatic metabolomics, and hepatic transcriptomics, followed by qRT-PCR and Western blot validation. Compared with the HFD model group, high-dose FMM significantly reduced serum TC, TG, and LDL-C levels by 34.2%, 40.1%, and 49.5%, respectively, increased HDL-C by 46.6%, and decreased hepatic TG accumulation by 27.8%. The high-dose FMM group showed lipid-lowering effects similar to those observed in the simvastatin group, particularly in major serum lipid parameters. Compared with the HFD group, high-dose FMM partially restored gut microbial α-diversity and altered the abundance of several bacterial taxa, including Lactobacillus and Roseburia. Fecal and hepatic metabolomic analyses revealed coordinated changes in metabolites associated with lipid metabolism, bile acid metabolism, and bile secretion-related pathways after FMM intervention. Hepatic transcriptomic analysis showed that differentially expressed genes between the FMM and HFD groups were enriched in the PPAR signaling and bile secretion pathways, with increased expression of *PPARα*, *CPT1a*, *CYP7A1*, *ABCG5*, *ABCG8*, and *MRP2* mRNAs and their corresponding proteins confirmed by qRT-PCR and Western blot analysis. Collectively, FMM ameliorated HFD-associated dyslipidemia and hepatic lipid accumulation, which are accompanied by changes in gut microbial composition, fatty acid oxidation, and cholesterol-bile acid metabolism.

## 1. Introduction

Dyslipidemia is a common metabolic disorder and one of the major risk factors for cardiovascular disease. It is primarily characterized by elevated levels of total cholesterol (TC), triglycerides (TG), and low-density lipoprotein cholesterol (LDL-C) and is accompanied by reduced levels of high-density lipoprotein cholesterol (HDL-C) [1]. Maintaining optimal blood lipid levels through lifestyle modification and pharmacotherapy can effectively reduce CVD risk [2]. However, although statins and novel lipid-lowering drugs have demonstrated significant clinical efficacy, the treatment and control rates of dyslipidemia remain far lower than its prevalence [3]. In addition to pharmacological therapy, lifestyle interventions, including dietary modification and physical exercise, are essential for the management of dyslipidemia. Nevertheless, long-term adherence to lifestyle changes remains a major challenge, particularly among populations with high rates of obesity and metabolic syndrome [4]. Thus, it is essential to identify a safe, effective, and sustainable approach to prevent and improve dyslipidemia.

Fermented dairy products are complex food systems composed of the milk matrix, fermentation microorganisms, and their metabolites. Lactic acid bacteria fermentation can alter the chemical composition of milk through proteolysis and carbohydrate metabolism, leading to the formation of fermentation-associated metabolites, including organic acids, peptides, exopolysaccharides, and other low-molecular-weight compounds, which may contribute to the nutritional and functional properties of fermented dairy products [5]. Recent studies have shown that the metabolic effects of fermented dairy products may not result from a single factor but may be jointly influenced by the milk matrix, strain-specific characteristics, and fermentation-derived metabolites [6]. In high-fat diet models, yogurt consumption has been shown to affect the gut microbiota and hepatic metabolism and to maintain the levels of branched-chain hydroxy acids generated during milk fermentation [7]. Yogurt fermented with *Lacticaseibacillus rhamnosus HF01* improved serum lipid profiles and hepatic lipid accumulation in HFD-fed mice, accompanied by changes in the gut microbiota and butyrate levels [8]. Similarly, compound fermented milk containing *Lactiplantibacillus plantarum Y44* alleviated HFD-induced hepatic lipid accumulation and modulated the gut microbiota and short-chain fatty acid-related changes [9]. In addition to fermented cow’s milk products, the metabolic effects of non-bovine dairy products have received increasing attention. For example, goat’s milk and its fermented products have been reported to affect hepatic lipid metabolism and gut microbial composition in HFD models [10], while mare’s milk and fermented mare’s milk have been shown to modulate the gut microbiota and intestinal homeostasis in experimental animals [11]. However, compared with fermented cow’s milk products, studies on non-bovine fermented dairy products, particularly fermented mare’s milk, remain limited with respect to lipid metabolism, and their potential involvement in gut-liver metabolic regulation requires further investigation.

Mare’s milk is considered a promising alternative dairy source because of its unique nutritional composition. Compared with cow’s milk, the protein composition of mare’s milk more closely resembles that of human milk, with a relatively balanced casein-to-whey protein ratio and high levels of functional proteins, including lysozyme, lactoferrin, and immunoglobulins [12]. Mare’s milk also contains relatively low levels of β-lactoglobulin, which may contribute to improved digestibility [13]. Although its total fat content is low, mare’s milk is rich in polyunsaturated fatty acids, which account for approximately 26.5% of its total fatty acids, and it has relatively low atherogenic and thrombogenic indices [14]. In addition, mare’s milk is rich in lactose and oligosaccharides. These components may exert prebiotic effects by promoting the growth of beneficial bacteria and helping maintain intestinal microbial homeostasis [15]. Collectively, these nutritional characteristics make mare’s milk a promising substrate for the development of functional fermented dairy products.

Following fermentation by lactic acid bacteria and yeasts, lactose and proteins in mare’s milk undergo microbial transformation and hydrolysis, resulting in changes in its chemical composition, nutritional properties, sensory characteristics, and potential biological activities [16,17]. Previous studies have reported that fermented mare’s milk may contain various fermentation-associated metabolites, including organic acids, peptides, and volatile compounds; however, their composition and abundance may vary among different fermented products. Previous studies have reported that fermented mare’s milk can generate multiple bioactive peptides, including angiotensin-converting enzyme inhibitory peptides and dipeptidyl peptidase-IV inhibitory peptides [18,19], and may have the potential to modulate the gut microbiota and improve host metabolic health. It should be noted that the metabolic effects of fermented milk may be jointly influenced by the milk matrix, the fermentation strains, and fermentation-derived metabolites [20]. At present, research on the lipid-lowering effects of fermented mare’s milk remains limited, and its key bioactive components and underlying mechanisms have not been systematically elucidated. Therefore, further studies are needed to evaluate the biological effects of fermented mare’s milk and characterize the associated physiological changes, which may provide a foundation for subsequent identification of its key bioactive components and mechanisms.

Our research group previously isolated and screened four *Lactiplantibacillus plantarum strains-Ni2A*, *Ni2B*, *Ni2C*, and *Ni2D*-from traditional fermented mare’s milk collected in Xinjiang. These strains exhibited favorable probiotic properties, including strong acid and bile tolerance, bile salt hydrolase activity, and high in vitro cholesterol-removal capacity, with the mixed-strain combination showing greater cholesterol-lowering activity than the individual strains. Building on these findings, we further evaluated the in vivo lipid-lowering effects of an equal-ratio mixture of the four strains and demonstrated that it could ameliorate hyperlipidemia-associated dyslipidemia and hepatic lipid accumulation while modulating the composition of the gut microbiota and related metabolic functions [21]. However, whether these strains retain their beneficial effects when incorporated into a fermented mare’s milk matrix, and whether the complete fermented product is associated with coordinated changes in the gut microbiota, fecal metabolites, hepatic metabolites, and hepatic gene expression, remains unclear. Therefore, this study focused on the FMM as a whole, we used an HFD-induced dyslipidemia rat model to evaluate the lipid-lowering effects of FMM and investigate its associated gut-liver metabolic responses and potential pathways at multiple levels, including the gut microbiota, fecal and hepatic metabolites, and hepatic gene and protein expression. This study provides a scientific basis for developing dietary interventions for dyslipidemia. It also provides a reference for the high-value utilization of mare’s milk as a local characteristic dairy resource.

## 2. Materials and Methods

### 2.1. Preparation of FMM

The four *Lactiplantibacillus plantarum* strains were activated for three successive passages. The activated cultures were centrifuged at 1000× *g* for 5 min at 4 °C, washed, collected, and resuspended in sterile physiological saline.

Fresh mare’s milk (Purchased at Nanshan Ranch in Urumqi, Xinjiang, China) was filtered through a 100-mesh nylon sieve and homogenized at 60 °C under a pressure of 25 MPa. The milk was then pasteurized at 72 °C for 30 s and cooled to room temperature. Subsequently, the milk was inoculated at 6% (*v*/*v*) with a compound probiotic culture containing 1 × 10^8^ CFU/mL viable cells, which consisted of L. plantarum Ni2A, Ni2B, Ni2C, and Ni2D mixed in equal proportions. Fermentation was carried out at 30 °C for 22 h. At the end of fermentation, the fermented mare’s milk (FMM) had a pH of approximately 4.4, a titratable acidity of approximately 90 °T, and a viable cell count of at least 1 × 10^7^ CFU/mL. After fermentation, the samples were cooled to terminate fermentation and then stored at 4 °C for 12 h for post-ripening. FMM was prepared every three days, the same batch of FMM was used for daily gavage administration, and the viable cell count in FMM at the time of gavage was maintained at no less than 1 × 10^7^ CFU/mL.

### 2.2. Animal Experimental Design

Sixty six-week-old male SPF Sprague–Dawley rats weighing 180–200 g were purchased from the Animal Experimental Center of Xinjiang Medical University. The animals were housed at 20–26 °C and 40–70% relative humidity under a 12 h light/12 h dark cycle. The rats were randomly divided into six groups (*n* = 10 per group): normal control group (NC), model control group (MC), simvastatin control group (PC), and high-, medium-, and low-dose fermented mare’s milk groups (LPH, LPM, and LPL). The NC group received a standard normal diet, whereas the remaining groups were fed a high-fat diet (HFD) containing 40% fat, 20% fructose, and 2% cholesterol. After three weeks of dietary intervention, blood samples were collected from the tail vein to measure serum TC, TG, LDL-C, and HDL-C levels and to evaluate the establishment of the HFD-induced dyslipidemia model. Following model assessment, the animals received the corresponding gavage treatments according to their assigned groups. Except for the NC group, which was fed a normal diet, all other groups were fed a high-fat diet (HFD). The LPH, LPM, and LPL groups were administered fermented mare’s milk by gavage at doses of 10, 8, and 6 mL/kg, respectively. The PC group was gavaged with simvastatin at 8 mg/kg, while the NC and MC groups were gavaged with normal saline at 8 mL/kg. All treatments were administered once daily. The gavage volumes were not volume-matched across the FMM dose groups; rather, the administered volume represented the predefined FMM dose. The intervention lasted for seven weeks, and body weight changes were recorded weekly. At the end of the experiment, the rats were fasted overnight and anesthetized with sodium pentobarbital. Serum, liver, and fecal samples were collected and stored at −80 °C.

All experimental procedures were conducted in accordance with the National Guidelines for Laboratory Animal Welfare. The experimental animals were purchased from the Animal Experimental Center of Xinjiang Medical University. The study was approved by the Institutional Animal Care and Use Committee (IACUC) under approval number IACUC-JT-20250417-42.

### 2.3. Biochemical Parameter Analysis

Serum total cholesterol (TC), triglycerides (TG), high-density lipoprotein cholesterol (HDL-C), low-density lipoprotein cholesterol (LDL-C), alanine aminotransferase (ALT), and aspartate aminotransferase (AST), as well as hepatic TC and TG levels, were measured using commercial assay kits (Nanjing Jiancheng Bioengineering Institute, Nanjing, China) according to the manufacturer’s instructions. Six biological samples per group were analyzed.

### 2.4. Liver Histopathological Analysis

Liver tissues were fixed in 4% paraformaldehyde, dehydrated through a graded ethanol series, and embedded in paraffin. Cut into 5-μm-thick sections. The sections were stained with hematoxylin and eosin (H&E) for histological observation. For quantitative analysis of hepatic fat vacuolization, H&E-stained liver sections were analyzed using ImageJ software (version 1.54g, National Institutes of Health, Bethesda, MD, USA). Representative non-overlapping microscopic fields were selected from each liver section under the same magnification. The total tissue area and the vacuolated area were measured after threshold adjustment, and large vessels, tissue tears, and obvious processing artifacts were excluded from the analysis. The same thresholding criteria were applied to all images. The percentage of fat vacuolization area was calculated as follows: fat vacuolization area (%) = vacuolated area/total tissue area × 100%.

### 2.5. Gut Microbiota Analysis

Gut microbiota profiling was performed by 16S rRNA gene sequencing. Total DNA was extracted from fecal samples using the OMEGA Soil DNA Kit (D5635-02; Omega Bio-Tek, Norcross, GA, USA). The quality and concentration of the extracted DNA were assessed by 0.8% agarose gel electrophoresis and Nanodrop spectrophotometry. The V3-V4 region of the bacterial 16S rRNA gene was amplified using the primers 338F (5′-ACTCCTACGGGAGGCAGCA-3′) and 806R (5′-GGACTACHVGGGTWTCTAAT-3′). The amplified products were subjected to paired-end sequencing on the Illumina NovaSeq 6000 platform (Illumina, San Diego, CA, USA). Raw sequencing data were processed using Cut adapt (v4.9) to generate amplicon sequence variants (ASVs). Gut microbiota composition was further analyzed based on alpha diversity indices, principal coordinate analysis (PCoA), non-metric multidimensional scaling (NMDS), and linear discriminant analysis effect size (LEfSe). The assay was performed by Shanghai Biotree Biotech Co., Ltd. (Shanghai, China).

### 2.6. Untargeted Metabolomics Analysis of Feces and Liver

We used untargeted metabolomics to further investigate the effects of FMM on the metabolite profiles of the gut and liver. Briefly, 25 mg of each sample was extracted with 500 μL of methanol/acetonitrile/water solution (2:2:1, *v*/*v*/*v*), followed by vortexing, homogenization, ultrasonication in an ice-water bath, and incubation at −40 °C. After filtration, equal aliquots of all samples were pooled to prepare quality control (QC) samples. Chromatographic separation was performed using a Vanquish ultra-high-performance liquid chromatography system (Thermo Fisher Scientific, Waltham, MA, USA). Polar metabolites were separated on a Waters ACQUITY UPLC BEH column (2.1 mm × 100 mm, 1.7 μm) using water containing 25 mmol/L ammonium acetate and 25 mmol/L ammonia solution as mobile phase A and acetonitrile as mobile phase B, whereas non-polar metabolites were separated on a Phenomenex Kinetex C18 column (2.1 mm × 100 mm, 2.6 μm) using water containing 0.01% acetic acid as mobile phase A and isopropanol/acetonitrile (1:1, *v*/*v*) as mobile phase B. The autosampler temperature was maintained at 4 °C, and the injection volume was 2 μL. MS and MS/MS data were acquired using an Orbitrap Exploris 120 mass spectrometer controlled by Xcalibur software (version 4.4, Thermo Fisher Scientific, Waltham, MA, USA) in both positive and negative ion modes. The main MS parameters were as follows: sheath gas flow rate, 50 Arb; auxiliary gas flow rate, 15 Arb; capillary temperature, 320 °C; full MS resolution, 60,000; MS/MS resolution, 15,000; collision energy, stepped normalized collision energy of 20/30/40; spray voltage, 3.8 kV in positive ion mode and −3.4 kV in negative ion mode [22]. Metabolites were considered significantly different when VIP > 1 and *p* < 0.05. The assay was performed by Shanghai Biotree Biotech Co., Ltd. (Shanghai, China).

### 2.7. Transcriptomics Analysis of Liver

Liver transcriptome sequencing was performed to further explore the molecular changes induced by FMM treatment. Total RNA was extracted from liver tissues of rats in the same group and subsequently sequenced on the Illumina NovaSeq X Plus PE150 platform. Raw sequencing data were filtered using fastp (v0.23.4) to obtain clean data. The paired-end clean reads were aligned to the reference genome using HISAT2. Based on the alignment results, transcripts were reconstructed using StringTie2 (v2.1.4), and the expression levels of all genes in each sample were calculated and normalized as fragments per kilobase of transcript per million mapped reads (FPKM). Principal component analysis (PCA) was performed based on the FPKM values of gene expression in all samples using the prcomp function in R to evaluate the relationships among samples. Differential expression analysis was performed using DESeq2 (v1.38.3) based on the gene-level read-count matrix. Differentially expressed genes were identified using |log2FC| > 1 and padj < 0.05. The assay was performed by Shanghai Biotree Biotech Co., Ltd.

### 2.8. Real-Time Quantitative Polymerase Chain Reaction (qRT-PCR)

Total RNA was extracted from liver samples and reverse-transcribed into cDNA. Target gene fragments were amplified using gene-specific primers, and the primer sequences are provided in the Supplementary Materials. Using *GAPDH* as the reference gene, relative expression levels were calculated using the 2^−ΔΔCt^ method.

### 2.9. Western Blot Analysis

Total liver protein was extracted using RIPA lysis buffer. The protein samples were separated by sodium dodecyl sulfate-polyacrylamide gel electrophoresis and subsequently transferred onto PVDF membranes. The membranes were blocked with 5% nonfat milk for 2 h and then washed three times with TBST for 5 min each. The membranes were incubated with primary antibodies overnight at 4 °C, followed by three washes with TBST for 5 min each. Subsequently, the membranes were incubated with secondary antibodies at room temperature for 1 h. After washing with TBST, the protein signals were detected by chemiluminescence exposure. GAPDH was used as the internal reference protein. Protein signals were detected using an Azure 600 imaging system (Azure Biosystems, Inc., Dublin, CA, USA) and quantified using ImageJ software.

### 2.10. Statistical Analysis

Statistical analyses were performed using SPSS 27.0 software (IBM Corp., Armonk, NY, USA). Data are presented as mean ± SD. A significance level of *p* < 0.05 was employed. Before one-way ANOVA, data normality and homogeneity of variance were assessed using the Shapiro–Wilk test and Levene’s test, respectively. For data meeting these assumptions, differences among multiple groups were evaluated by one-way analysis of variance (ANOVA) combined with Tukey’s HSD post-hoc test. When the assumptions for parametric tests were not met, the Kruskal–Wallis H test was used. The data visualization was achieved using Origin 2022.

## 3. Results

### 3.1. Effects of FMM on Body Weight and Lipid Metabolism in Rats Induced by HFD

**Figure 1:**
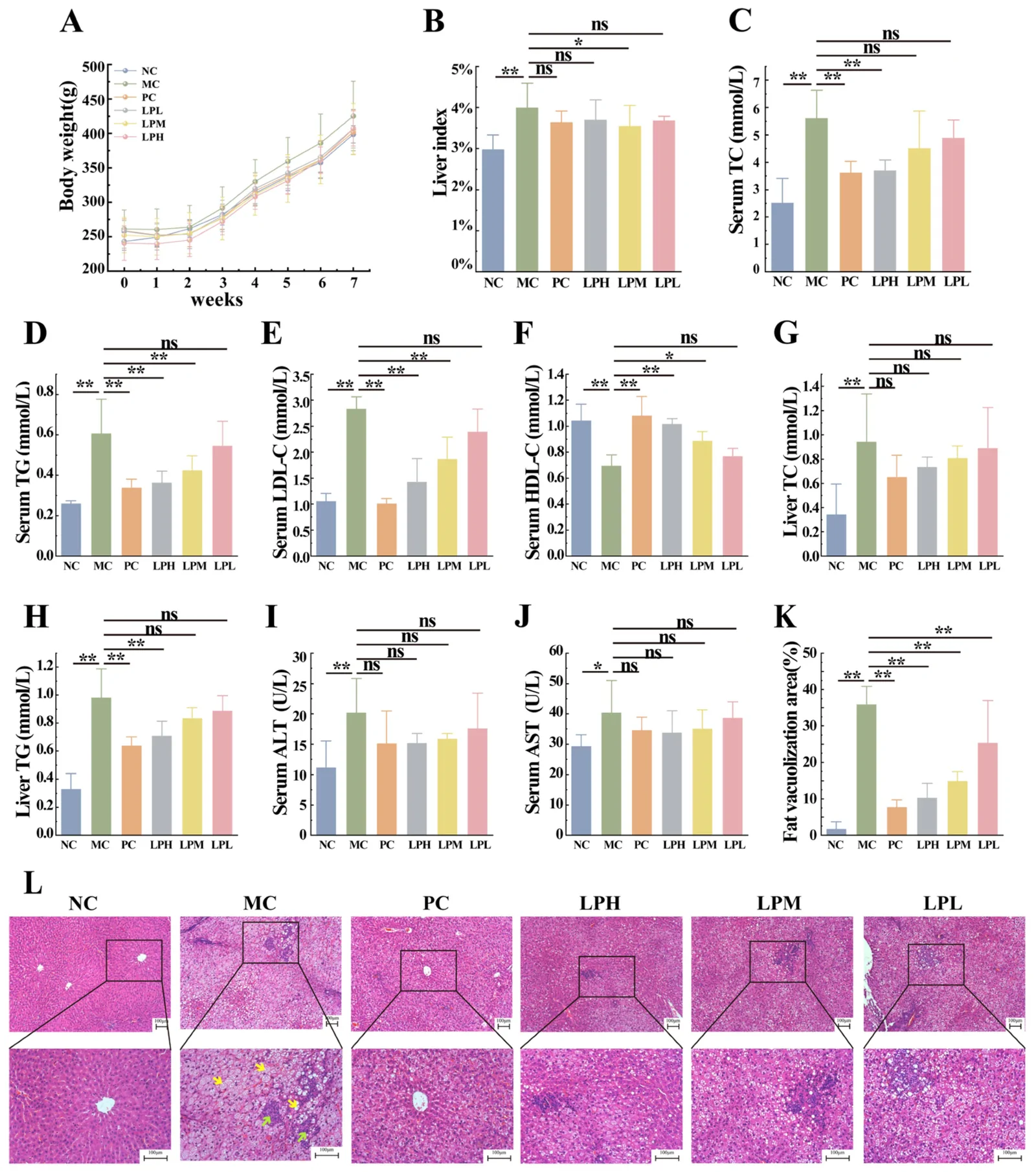
FMM ameliorated HFD-induced lipid metabolism disorders and lipid deposition in rats. (**A**) Body weight during the intervention period (*n* = 10); (**B**) Liver index; (**C**–**F**) Serum TC, TG, LDL-C, HDL-C levels; (**G**,**H**) Hepatic TC, TG levels; (**I**,**J**) Serum ALT, AST activities; (**K**) Fat vacuolization area; (**L**) H&E staining of liver tissues (Yellow arrows indicate cytoplasmic vacuolization, and green arrows indicate inflammatory responses.). * *p* < 0.05; ** *p* < 0.01, ns, not significant. sample size (*n*) = 6.

Body weight increased progressively over the 7-week intervention period in all groups (Figure 1A). Repeated-measures ANOVA showed a significant effect of time (*p* < 0.001), whereas neither the group effect nor the time × group interaction was significant *(p* = 0.468 and *p* = 0.087, respectively). The liver index reflects the degree of hepatomegaly induced by HFD, and Figure 1B shows that, compared with the MC group, the LPM group had a significantly lower liver index, while the other treatment groups also showed slight decreases that were not statistically significant. Serum biochemical parameters were also measured, and the results (Figure 1C–H) showed that FMM intervention significantly reduced serum TC, TG, and LDL-C levels (*p* < 0.05), while HDL-C showed an increasing trend. Compared with the MC group, the LPH group had a significantly lower hepatic TG content (*p* < 0.01), whereas its hepatic TC content did not differ significantly. In addition, serum AST and ALT activities were measured (Figure 1I,J), the results showed that serum AST and ALT activities were lower in all FMM-treated groups and the PC group than in the MC group, but the differences were not statistically significant. This suggests that FMM had a limited hepatoprotective effect and did not effectively alleviate HFD-induced hepatocellular injury during the intervention period.

H&E staining was performed to evaluate the effects of FMM on liver histopathology (Figure 1L). Hepatocyte morphology in the NC group was normal. The MC group showed disorganized hepatic cord arrangement, excessive lipid droplet accumulation, cytoplasmic vacuolization (Figure 1K), as indicated by the yellow arrows, and inflammatory responses, as indicated by the green arrows. These results suggest that HFD impaired hepatic lipid transport and metabolic function, leading to severe liver injury, which was consistent with the changes in AST and ALT activities. After FMM intervention, hepatic steatosis and cytoplasmic vacuolization were reduced, and hepatocellular inflammation was also alleviated in a dose-dependent manner. Therefore, the LPH group, which showed the greatest improvement, was selected for subsequent mechanistic analysis.

### 3.2. Effects of FMM on the Gut Microbiota

**Figure 2:**
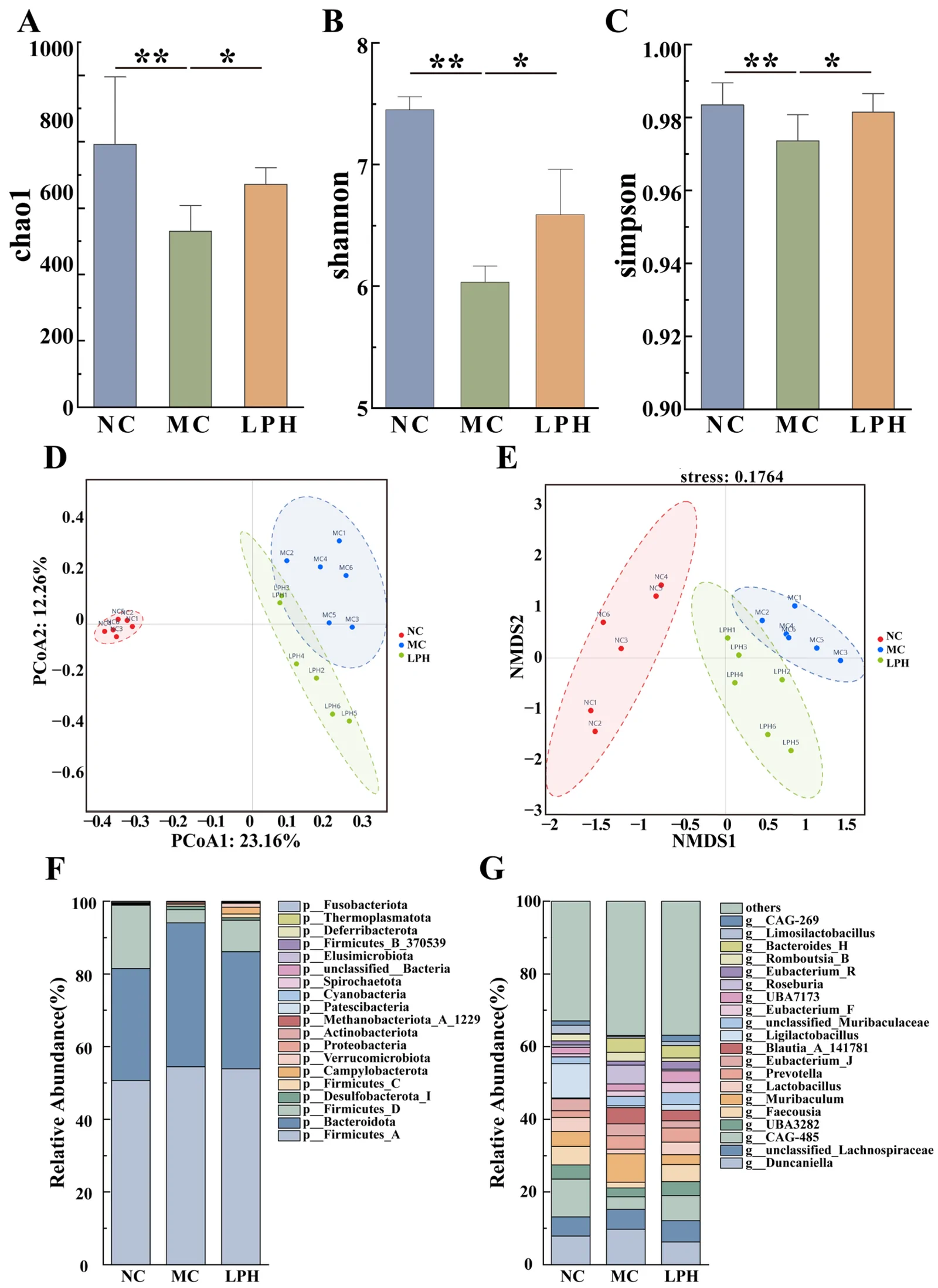
FMM reshaped the gut microbiota structure. (**A**–**C**) α-Diversity of the gut microbiota; (**D**) PCoA plot; (**E**) NMDS plot; (**F**,**G**) Relative abundances of the gut microbiota at the phylum and genus levels. * *p* < 0.05; ** *p* < 0.01, *n* = 6.

HFD can alter the gut microbiota, and these microorganisms and their metabolites may enter the liver through the portal circulation and bile acid enterohepatic circulation, thereby affecting lipid metabolism-related processes [23,24]. The 16S rRNA sequencing results demonstrated that the Chao1, Shannon, and Simpson indices were significantly reduced in the MC group (*p* < 0.01), whereas FMM intervention significantly restored the α-diversity indices in the LPH group (*p* < 0.05). These results indicate that FMM effectively alleviated the HFD-induced reduction in gut microbiota α-diversity (Figure 2A–C). PCoA and NMDS analyses showed that HFD markedly altered the structure of the gut microbiota. The LPH group showed limited overlap with the MC group and exhibited a tendency toward separation, suggesting that FMM intervention modulated the HFD-associated gut microbial community (Figure 2D,E). At the phylum level (Figure 2F), Firmicutes and Bacteroidota were the dominant phyla in all groups, suggesting that FMM intervention primarily affected the genera belonging to these two phyla. At the genus level (Figure 2G), LPH intervention significantly increased the relative abundances of Lactobacillus and Roseburia, both of which are well-recognized beneficial gut bacteria. These results indicate that FMM can modulate gut microbiota and improve gut microbial dysbiosis.

LEfSe analysis was performed to identify bacterial taxa differentially enriched among the groups. As shown in Figure S1A,B, several bacterial genera enriched in the NC group, including *Lactococcus* and *Lactobacillus*, constituted the core microbial community associated with a healthy gut. However, these health-related bacteria were markedly reduced in the MC group, which may have contributed to the development of gut microbiota dysbiosis. In contrast, the LPH group was significantly enriched with multiple beneficial bacteria. In particular, the recovery of the mucin-degrading bacterium *Akkermansia* and several short-chain fatty acid (SCFA)-producing bacteria was a key feature indicating a shift in gut microbial composition after FMM intervention. Combined with the MetagenomeSeq analysis (Figure S1D) and LEfSe results, 10 genera were selected for Spearman correlation analysis with serum lipid parameters (Figure S1B). The results showed that *Ligilactobacillus*, *Copromonas*, *Negativibacillus*, *NM07-P-09*, and *Phocea* were strongly correlated with serum lipid parameters, suggesting that these genera may be regulated by FMM.

### 3.3. Effects of FMM on Fecal and Liver Metabolomes

**Figure 3:**
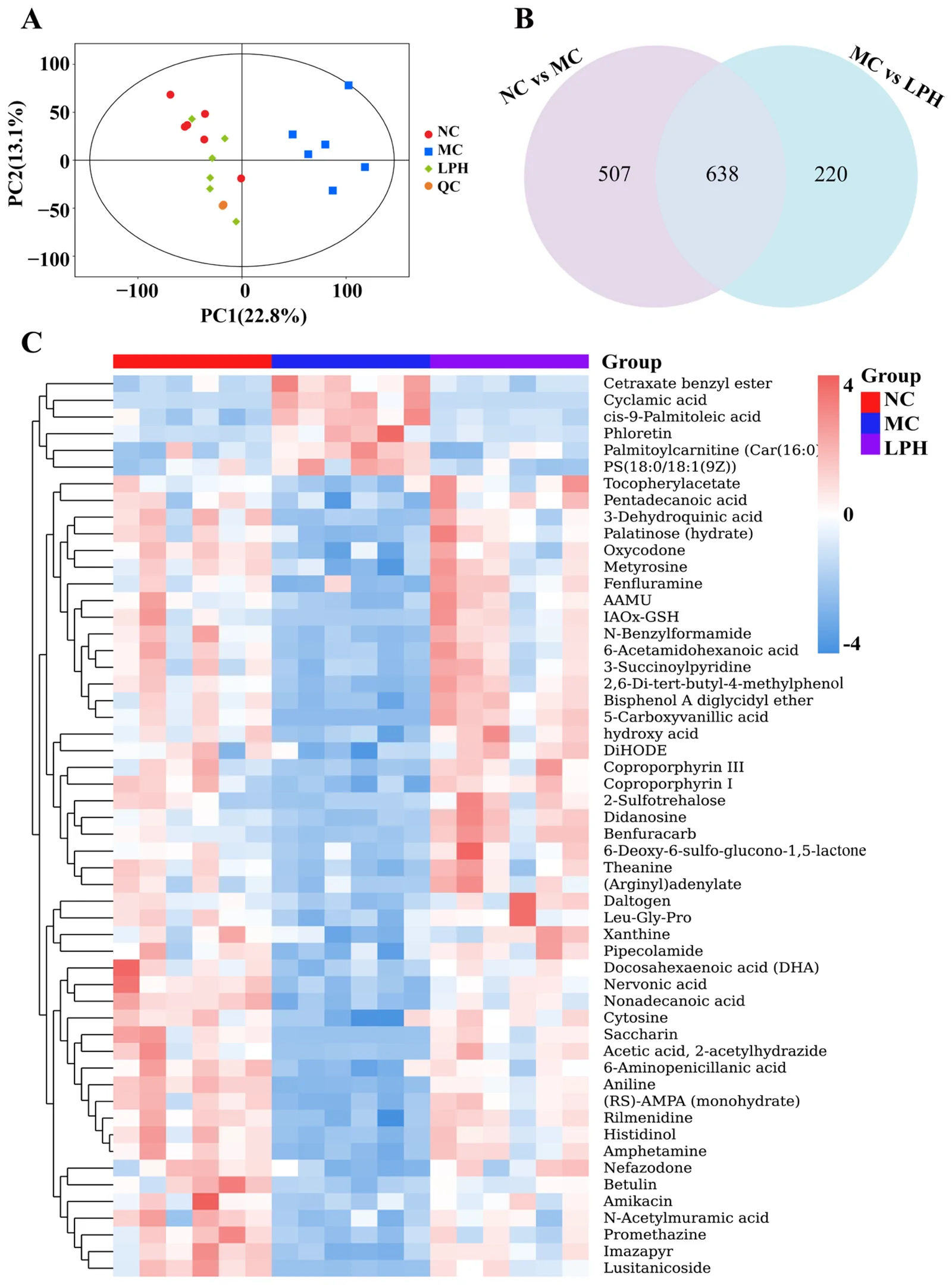
Effects of FMM on fecal metabolites. (**A**) PCA score plot; (**B**) Venn diagram of differential metabolites from the two comparisons and (**C**) heatmap of overlapping metabolites; *n* = 6.

**Figure 4:**
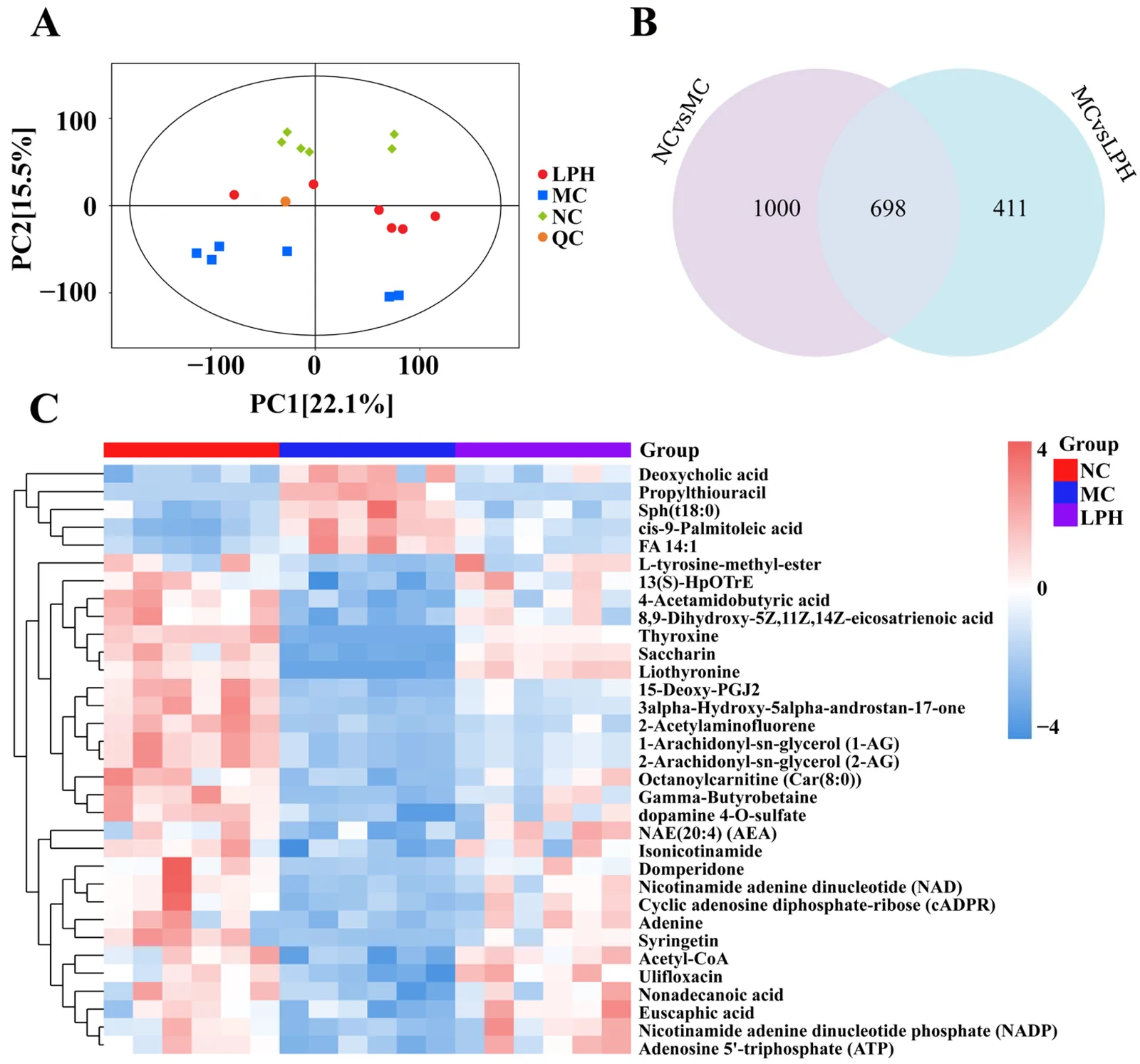
Effects of FMM on hepatic metabolites. (**A**) PCA score plot; (**B**) Venn diagram of differential metabolites from the two comparisons and (**C**) heatmap of overlapping metabolites, *n* = 6.

To characterize the impact of FMM intervention on the metabolite profiles of rats, fecal and liver metabolites were analyzed using UHPLC-Orbitrap-MS/MS. Untargeted metabolomic analysis showed that FMM markedly altered the fecal and hepatic metabolic profiles of HFD-fed rats. Principal component analysis (PCA) revealed a clear separation of liver and fecal metabolites between the MC and NC groups, indicating that HFD induced substantial changes in the fecal and hepatic metabolite profiles of rats (Figure 3A and Figure 4A). After FMM intervention, the fecal metabolite profile of the LPH group almost overlapped with that of the NC group, while the hepatic metabolite profile also showed a tendency to shift toward the NC group, suggesting that FMM partially restored the metabolic profiles disrupted by HFD feeding.

**Figure 5:**
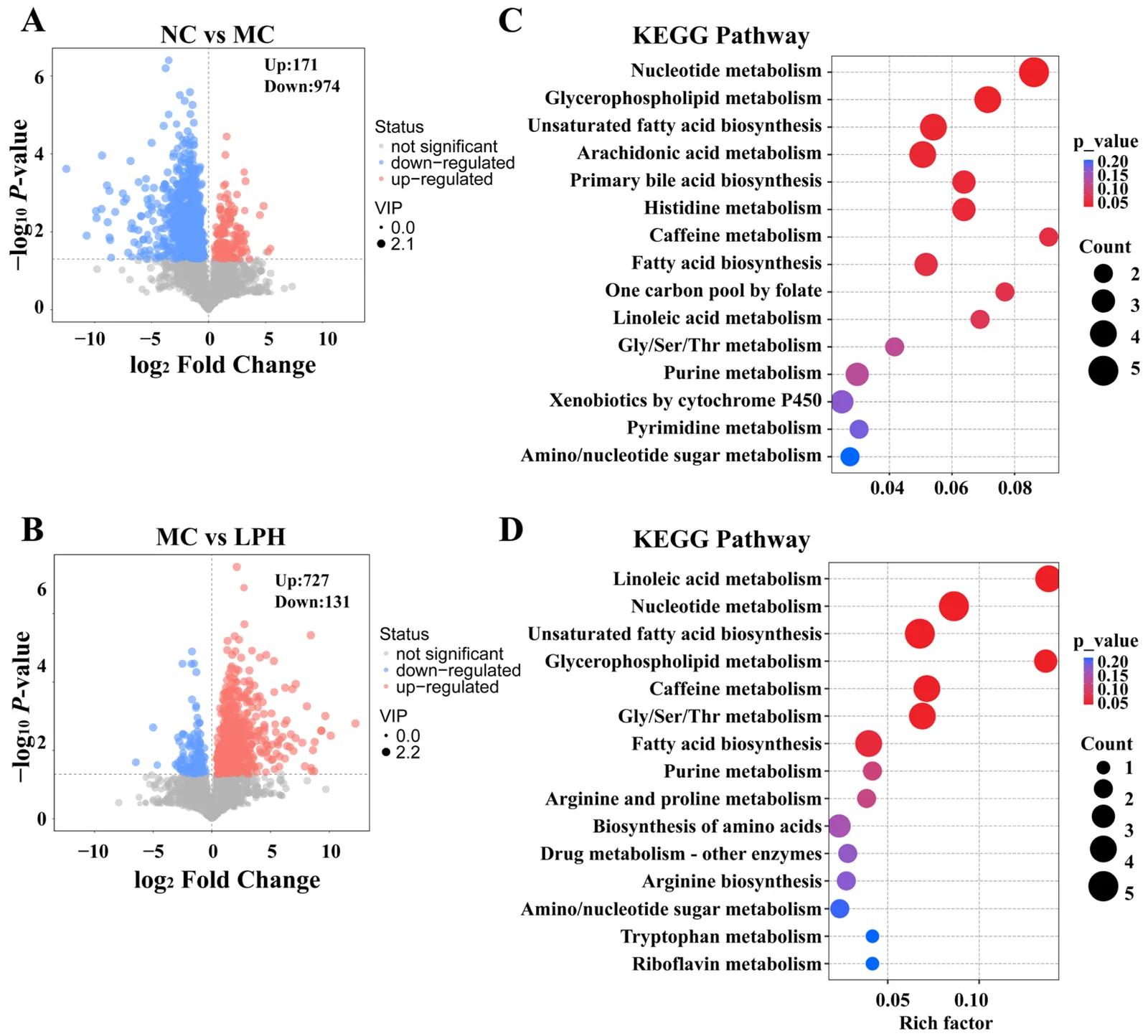
Effects of FMM on fecal metabolites. (**A**,**B**) Volcano plots of NC vs. MC and MC vs. LPH; (**C**,**D**) KEGG pathway enrichment bubble plots of NC vs. MC and MC vs. LPH, *n* = 6.

**Figure 6:**
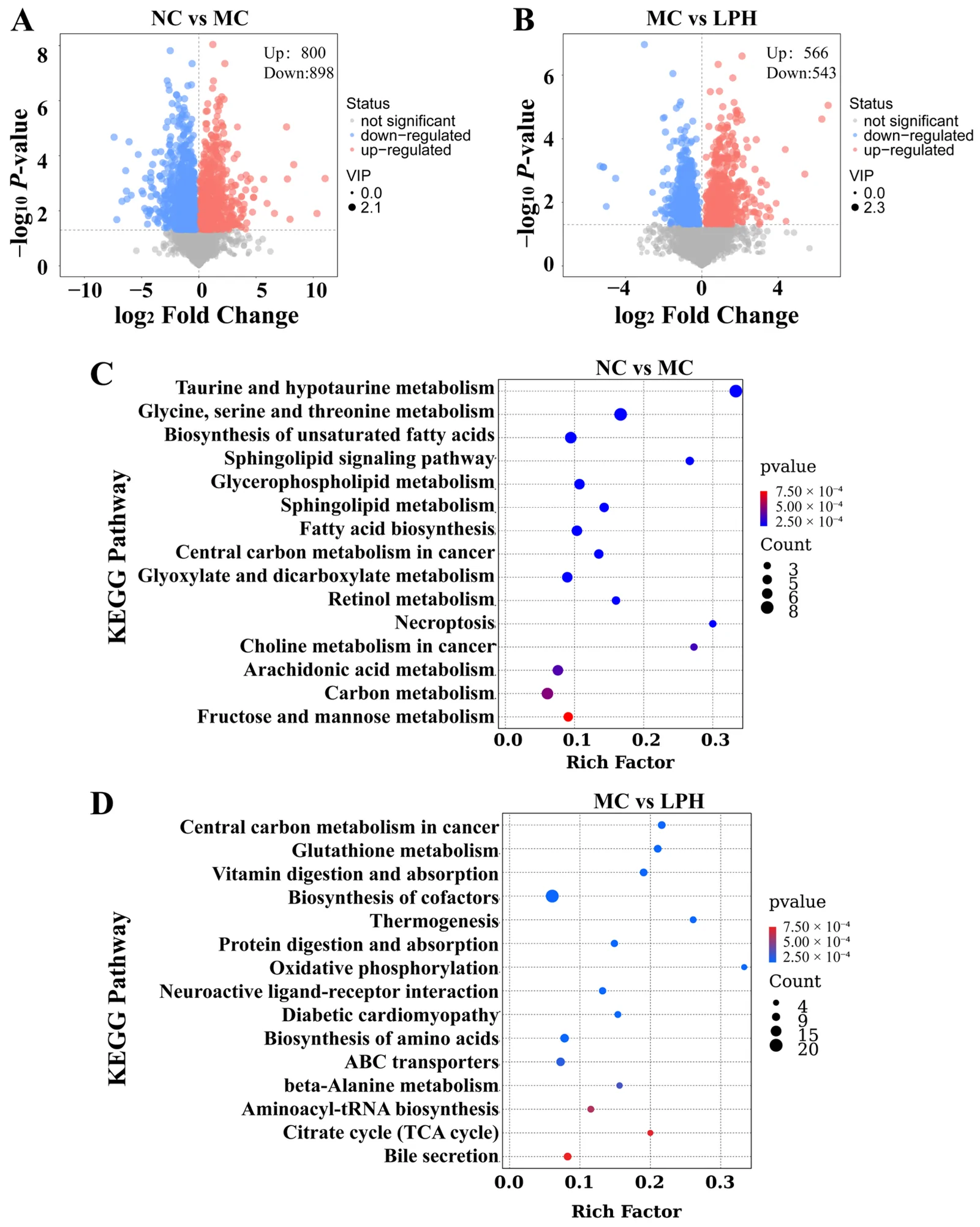
Effects of FMM on hepatic metabolites. (**A**,**B**) Volcano plots of NC vs. MC and MC vs. LPH; (**C**,**D**) KEGG pathway enrichment bubble plots of NC vs. MC and MC vs. LPH, *n* = 6.

Analysis of the differential metabolites identified in the two pairwise comparisons revealed 638 common differential metabolites in fecal samples and 698 common differential metabolites in liver samples (Figure 3B and Figure 4B). These metabolites mainly belonged to amino acids and their derivatives, as well as lipid compounds. After further screening based on |log2FC| > 1, the selected metabolites were subjected to heatmap analysis (Figure 3C and Figure 4C). The results showed clear metabolic differences between the MC and LPH groups in both feces and liver. The differential metabolites in feces were mainly associated with bile acid metabolism, amino acid metabolism, fatty acid metabolism, and exogenous or diet-derived compounds, suggesting that LPH may affect gut microbiota-related metabolic processes. In the liver, the differential metabolites were primarily involved in lipid oxidation, energy metabolism, NAD-related redox metabolism, and inflammatory lipid mediators, indicating that LPH may further regulate hepatic metabolic function. The metabolites shared between the two tissues, such as deoxycholic acid, nonadecanoic acid, acetyl-CoA, and nicotinamide/NAD-related compounds, suggest that these metabolic differences may be closely associated with enterohepatic circulation of bile acids, fatty acid oxidation, and energy metabolism, reflecting potential differences in gut–liver axis metabolic regulation between the MC and LPH groups. The upregulated metabolites were significantly negatively correlated with TC, TG, and LDL-C, whereas the downregulated metabolites were positively correlated with these lipid parameters (Figure S2A,B), indicating that these metabolites were closely associated with blood lipid levels. To visualize the overall upregulation and downregulation patterns of differential metabolites in each comparison, volcano plots were generated for each group comparison (Figure 5A,B and Figure 6A,B). The results indicated that both HFD induction and FMM intervention markedly changed the metabolite composition in feces and liver.

KEGG pathway enrichment analysis was performed to further characterize changes in host metabolism after FMM intervention. Differential metabolites between the NC and MC groups were mainly enriched in lipid metabolism-related pathways. (Figure 5C and Figure 6C), suggesting that HFD induced abnormal alterations in lipid metabolism-related pathways. After FMM intervention, differential metabolites in feces between the MC and LPH groups were enriched in primary bile acid metabolism, while differential metabolites in the liver were enriched in bile secretion. The enrichment of these two pathways suggests that FMM intervention may primarily affect host cholesterol conversion and metabolism. (Figure 5D and Figure 6D), indicating that FMM intervention may gradually improve HFD-induced lipid metabolic disorders.

### 3.4. Effects of FMM on the Liver Transcriptome

**Figure 7:**
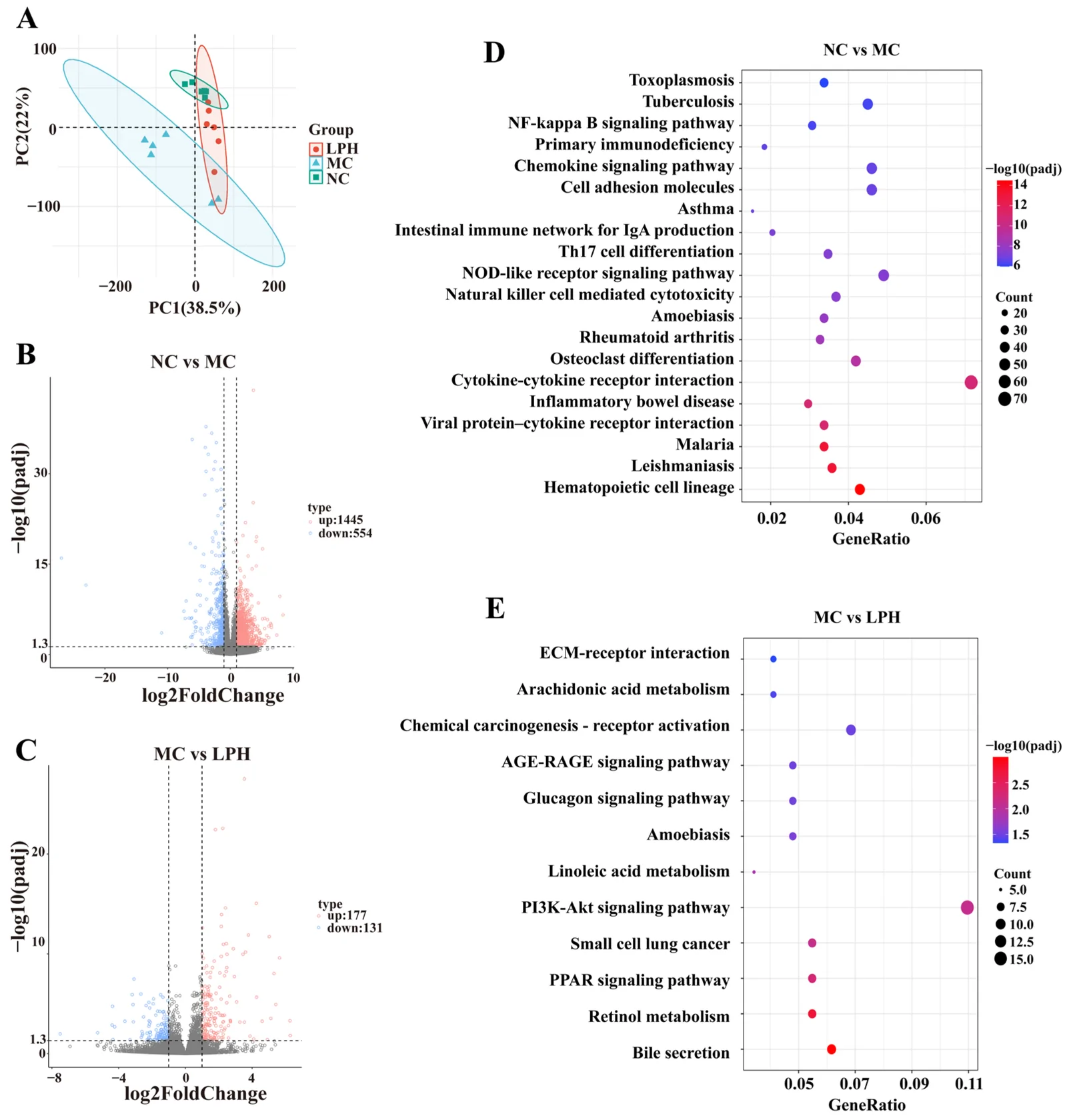
Effects of FMM on hepatic gene expression. (**A**) PCA score plot; (**B**) Volcano plot of the NC vs. MC comparison; (**C**) Volcano plot of the MC vs. LPH comparison; (**D**) KEGG pathway enrichment bubble plot of differentially expressed genes between the NC and MC groups; (**E**) KEGG pathway enrichment bubble plot of differentially expressed genes between the MC and LPH groups, *n* = 6.

Transcriptomic analysis of liver tissue was performed to further clarify the molecular mechanism underlying the lipid-lowering effect of FMM. The PCA results demonstrated a separation trend among the NC, MC, and LPH groups (Figure 7A), indicating differences in gene expression profiles among the groups. This result was further supported by the volcano plots (Figure 7B,C). Compared with the NC group, HFD induction resulted in 1445 upregulated and 554 downregulated differentially expressed genes in the MC group. After FMM intervention, the LPH group also showed 177 upregulated and 131 downregulated differentially expressed genes compared with the MC group (|log2FC| > 1, *p* < 0.05), suggesting that FMM could modulate the hepatic gene expression profile of HFD-fed rats.

**Figure 8:**
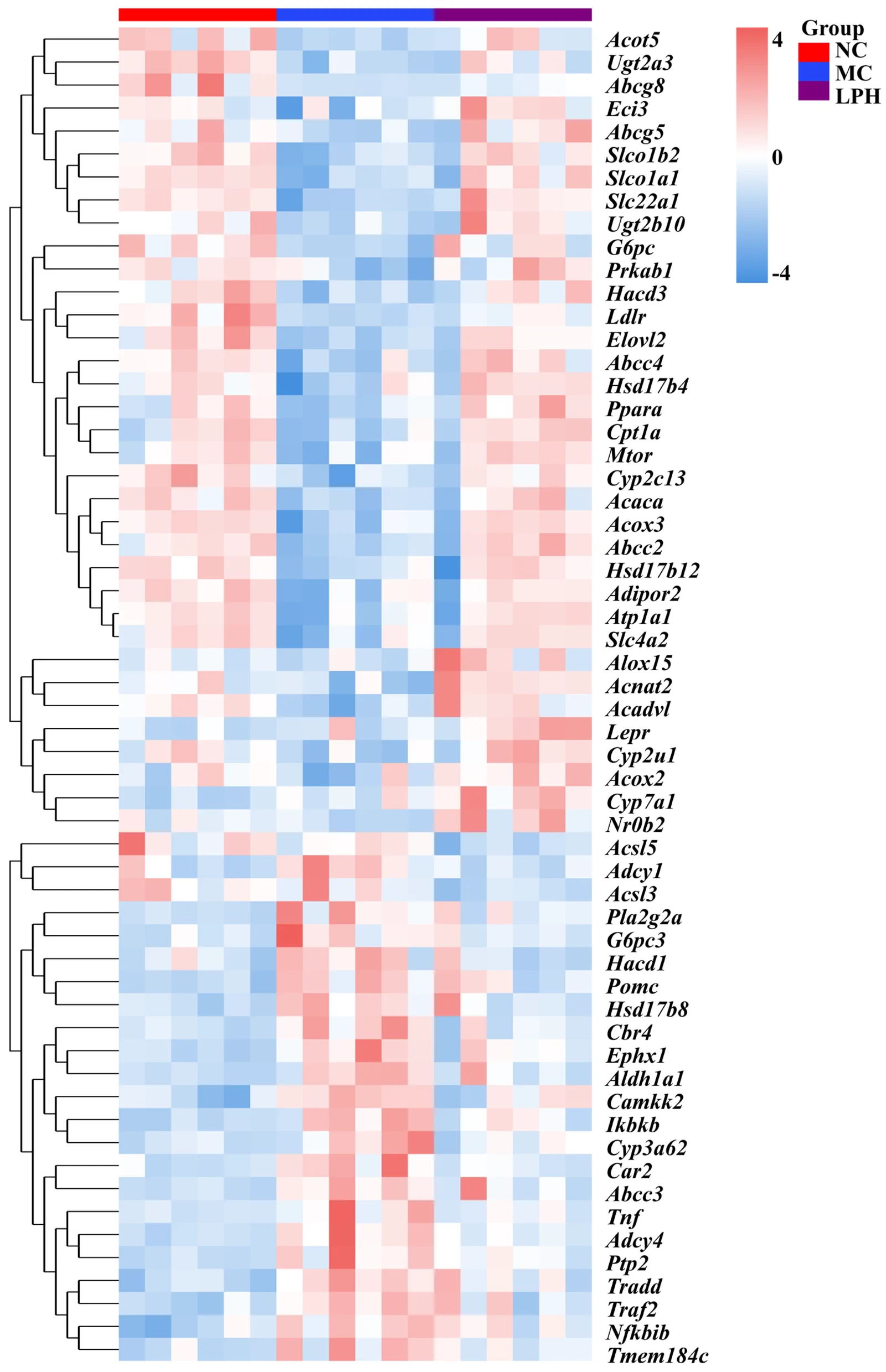
Effects of FMM on hepatic gene expression. Heatmap of differentially expressed genes, *n* = 6.

To determine the pathway enrichment of the differentially expressed genes, KEGG analysis was performed. The DEGs between the NC and MC groups were significantly enriched in the *NF-κB* signaling pathway and cell adhesion molecules, which are closely associated with inflammatory responses and lipid deposition (Figure 7D). After FMM intervention, the DEGs were significantly enriched in bile secretion, linoleic acid metabolism, and the PPAR signaling pathway, showing that FMM treatment was associated with changes in hepatic gene-expression profiles related to lipid metabolism (Figure 7E). The differentially expressed genes involved in the pathways shown in Figure 7E were further analyzed using a heatmap, as shown in Figure 8.

After FMM intervention, transcriptomic analysis of liver tissue revealed that differentially expressed genes were significantly enriched in the bile secretion and *PPAR* signaling pathways. In addition, metabolomic enrichment analysis of hepatic differential metabolites indicated enrichment in bile secretion pathways, while fecal differential metabolites were enriched in the primary bile acid biosynthesis pathway. Combined with the enrichment of multiple lipid metabolism-related pathways in metabolomics data, *PPARα*, as an upstream nuclear regulatory factor, is known to regulate the expression of *CYP7A1* and *CPT1a*. Therefore, it can be inferred that the lipid-lowering effects of FMM were accompanied by changes in two pathway-related gene sets: (1) *PPARα*/*CPT1a*-mediated fatty acid β-oxidation, and (2) *PPARα*/*CYP7A1*-mediated cholesterol conversion and efflux.

**Figure 9:**
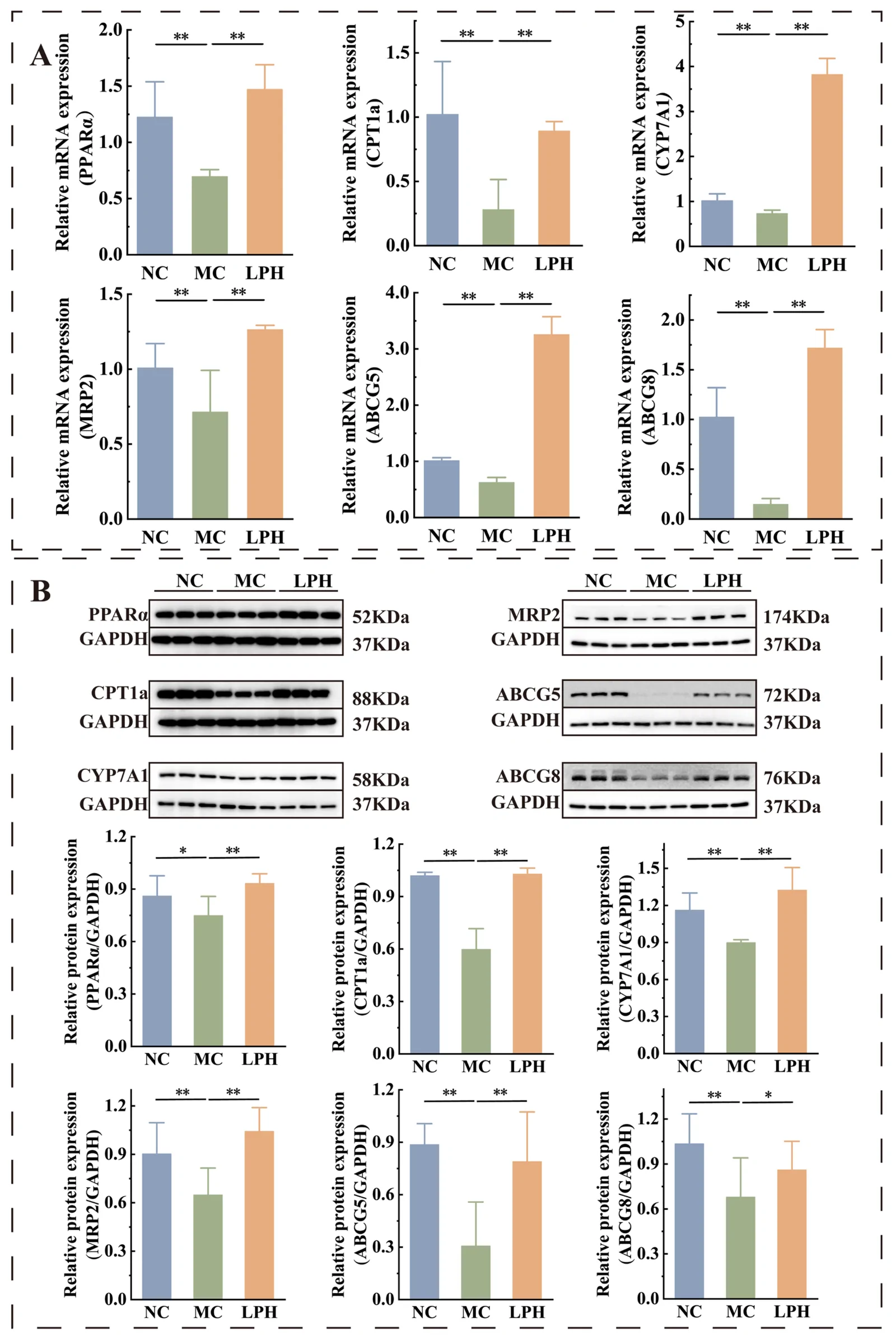
(**A**) The liver mRNA expression of *PPARα*, *CYP7A1*, *ABCG5*, *ABCG8*, *CPT1a* and *MRP2*. (**B**) The liver protein expression of PPARα, CYP7A1, ABCG5, ABCG8, CPT1a and MRP2, * *p* < 0.05; ** *p* < 0.01 *n* = 6.

P*PARα*, *CYP7A1*, *ABCG5/G8*, *CPT1a*, and *MRP2* exhibit an upregulation trend and are predominantly localized in the *PPARα* signaling pathway and bile secretion. *PPARα* is an upstream nuclear signaling transcription factor for both *CYP7A1* and *CPT1a. CYP7A1* accelerates the conversion of cholesterol into bile acids within hepatocytes, while *ABCG5/G8* and *MRP2* facilitate the excretion of cholesterol and bile acids from hepatocytes; *CPT1a* serves as a key factor in fatty acid β-oxidation. Western blotting (WB) and qRT-PCR validation demonstrated that the expression levels of these were significantly upregulated (Figure 9).

## 4. Discussion

HFD-induced lipid metabolism disorders involve peripheral dyslipidemia, hepatic lipid deposition, and impaired enterohepatic circulation of cholesterol. In the present study, probiotic-fermented mare’s milk (FMM) was prepared using cholesterol-lowering probiotics isolated from traditional fermented mare’s milk as starter cultures. The lipid-lowering effects and associated gut-liver metabolic responses were investigated from multiple perspectives, including blood lipid phenotypes, gut microbiota, fecal metabolomics, hepatic metabolomics, hepatic transcriptomics, and validate the key genes and proteins.

In the present study, FMM intervention increased the relative abundances of *Ligilactobacillus*, *Copromonas*, and *NM07-P-09*. These genera showed correlations with blood lipid parameters, suggesting that they may be associated with the metabolic alterations observed after FMM intervention. The increase in *Ligilactobacillus* suggests that this genus may be an FMM-responsive taxon associated with cholesterol-related phenotypes. In an HFD model, *Lactobacillus reuteri Fn041* was reported to reduce blood fat levels and increase fecal cholesterol and bile acid excretion, further supporting the mechanism by which *lactobacilli* improve lipid homeostasis through cholesterol-bile acid metabolism [25]. *Copromonas* and *NM07-P-09* may jointly reflect the regulatory effect of FMM on intestinal anaerobic metabolic bacteria. *Copromonas* belongs to the family *Lachnospiraceae*, an important group of strictly anaerobic fermentative bacteria in the gut that participates in the utilization of complex carbon sources [26,27]. The enrichment of *NM07-P-09* after FMM intervention suggests that it may be an important responsive taxon involved in FMM-mediated regulation of the gut microbial ecosystem. Previous studies have also observed an association between *NM07-P-09* and improved intestinal status after intervention. Qiu et al. reported that *NM07-P-09* was significantly decreased in mice with LPS-induced intestinal injury [28], whereas polysaccharide intervention alleviated intestinal injury, oxidative stress, and inflammatory responses and modulated differential bacterial taxa, including *NM07-P-09*. The increase in *NM07-P-09* abundance after FMM intervention was consistent with the trend reported in that study. *NM07-P-09* belongs to the phylum Actinobacteriota, which has been linked to bile acid, cholesterol, and lipid metabolism [29,30]. In a word, changes in the gut microbiota may provide microbiological evidence supporting a possible association between FMM-induced microbiota shifts and fatty acid oxidation- and cholesterol excretion-related pathways.

Fecal and hepatic metabolomic analyses further revealed coordinated alterations in gut-liver axis-related lipid metabolism after FMM intervention. The results showed that after FMM intervention, differential metabolites in both the fecal and liver metabolomes were commonly enriched in multiple lipid metabolism-related pathways. This result implies that the ameliorative influence of FMM on HFD-induced lipid metabolism disorders may be related to coordinated changes in the interaction between the intestinal metabolic environment and hepatic lipid metabolism. Previous studies have reported that dietary fat can affect host metabolism and hepatic fat deposition through gut microbiota-mediated mechanisms, and that dietary fat, gut microbiota, bile acid profiles, and hepatic lipid metabolism are closely interconnected [31]. Cai et al. also found that the metabolic profiles of the liver and cecal contents were jointly altered in HFD-induced obese mice [32], suggesting that intestinal and hepatic metabolites can collectively reflect HFD-induced metabolic disorders. We identified multiple fatty acids and fatty acid derivatives in the two metabolomic datasets. Among them, palmitoleic acid has been considered a lipid hormone-like molecule that can act as a metabolic signal between adipose tissue and distant organs [33]. After FMM intervention, the levels of Cer(d18:1/16:1) were decreased in both fecal and hepatic samples. Ceramide, an important lipotoxic lipid molecule, tends to accumulate abnormally under metabolic disorders such as obesity and dyslipidemia. A growing body of evidence has shown that ceramide can promote the onset and progression of metabolic diseases, including insulin resistance, hepatic steatosis, and cardiac metabolic dysfunction, by mediating multiple signaling pathways [34,35]. Therefore, the coordinated alteration of Cer(d18:1/16:1) in feces and liver suggests that FMM may not only affect fatty acid metabolism but also alleviate lipotoxic stress by regulating sphingolipid metabolism. Moreover, the coordinated regulation of these fatty acid metabolites by FMM reflects its systemic role in reducing lipid deposition and improving fatty acid metabolic imbalance.

The *PPARα-CPT1a* axis may represent an important pathway associated with the hepatic response to FMM intervention. *PPARα* can regulate fatty acid uptake as well as mitochondrial, peroxisomal β-oxidation and maintain hepatic energy metabolic homeostasis. In this study, expression levels of *PPARα* and *CPT1a* were upregulated after FMM intervention, suggesting that increased expression of fatty acid oxidation-related genes may accompany the reduction in hepatic TG accumulation after FMM intervention. This finding was also consistent with the decreased hepatic TC and TG levels, reduced lipid droplet deposition observed by H&E staining, and remodeling of fatty acid-related metabolites revealed by metabolomic analysis.

Changes in the *CYP7A1-MRP2-ABCG5/G8* axis suggest that cholesterol catabolism- and hepatobiliary excretion-related pathways were affected after FMM intervention. Pharmacological regulation of *CYP7A1* can affect bile acid synthesis, cholesterol clearance, and lipid homeostasis [36], suggesting that *CYP7A1* is a potential therapeutic target for hyperlipidemia and atherosclerosis. Meanwhile, the upregulation of *ABCG5* and *ABCG8* after FMM intervention further suggests that FMM may promote cholesterol efflux through the biliary pathway. *ABCG5/G8* is a heterodimer composed of two half-transporters and mainly mediates the efflux of cholesterol in hepatocytes and intestinal epithelial cells. Defects in the *ABCG5/G8* genes not only increase cholesterol levels in the blood and hepatocytes but also enhance the body’s sensitivity to dietary cholesterol [37]. The upregulation of *MRP2* further suggests that FMM may enhance the canalicular efflux and detoxification capacity of conjugated metabolites in the liver. *MRP2* is located on the canalicular membrane of hepatocytes and is mainly responsible for the biliary excretion of conjugated organic anions, bilirubin, and some bile acid-related conjugates. Dysfunction of *MRP2* is closely associated with cholestasis and jaundice [38]. In this study, the upregulation of these genes indicates that FMM may reduce cholesterol levels was accompanied by changes in genes related to cholesterol-to-bile-acid conversion and biliary efflux. Nevertheless, this study has several limitations. Because unfermented mare’s milk and strain-only control groups were not included, the independent contributions of the mare’s milk matrix, the fermentation process, and the *Lactiplantibacillus plantarum Ni2* consortium could not be distinguished. Therefore, the effects observed in the present study should be attributed to the complete FMM product rather than to any individual component or process. Further studies using appropriate comparator groups are needed to clarify the specific contributions of the milk matrix, fermentation, and probiotic strains. In addition, the specific chemical constituents responsible for the observed biological effects of FMM were not identified in the present study. Although fermentation-associated metabolites have been reported in fermented dairy products, whether the active components originate from the original mare’s milk matrix or are generated during fermentation remains unclear. Future studies combining comparative chemical profiling of unfermented and fermented mare’s milk with targeted functional validation are required to identify the key bioactive components.

**Figure 10:**
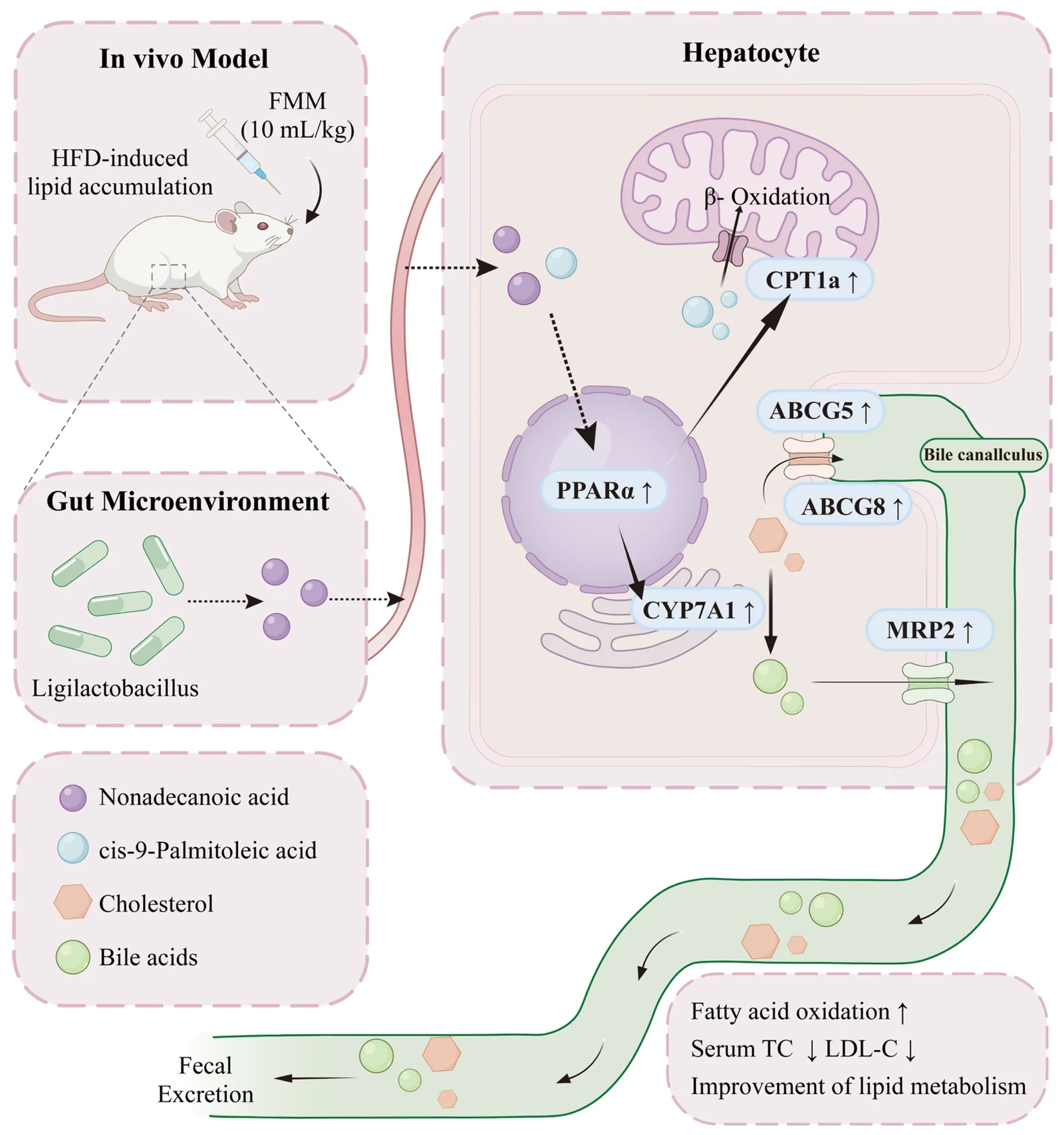
Schematic summary of gut-liver metabolic changes associated with FMM intervention in HFD-induced lipid accumulation. (Solid arrows indicate direct processes, whereas dashed arrows indicate indirect processes involving intermediate steps).

In summary, FMM intervention was associated with increased expression of *PPARα* and *CPT1a*, together with changes consistent with enhanced hepatic fatty acid β-oxidation and reduced hepatic lipid deposition. In parallel, FMM intervention was accompanied by increased expression of *CYP7A1*, *ABCG5*, *ABCG8*, and *MRP2*, suggesting alterations in cholesterol-to-bile-acid conversion, cholesterol efflux, and bile-related conjugate excretion pathways (Figure 10). The results showed that FMM intervention ameliorated lipid metabolism disorders in HFD-fed rats, accompanied by coordinated changes in the gut microbiota, gut-liver axis-related fatty acid metabolites, and hepatic cholesterol/fatty acid metabolic pathways.

## 5. Conclusions

This study showed that the complete probiotic-fermented mare’s milk (FMM) product alleviated HFD-induced dyslipidemia and hepatic lipid accumulation in rats. FMM reduced serum and hepatic lipid levels, alleviated liver injury, and was associated with modulation of the gut microbiota and coordinated changes in fecal and hepatic metabolites. In the high-dose group, multi-omics analyses and molecular validation indicated alterations in PPAR signaling, fatty acid β-oxidation, and cholesterol-bile acid metabolism, which are accompanied by increased expression of *PPARα*, *CPT1a*, *CYP7A1*, *ABCG5*, *ABCG8*, and *MRP2*. These findings support the potential of FMM as a functional fermented dairy food. However, because unfermented mare’s milk and probiotic-only controls were not included, the observed effects cannot be specifically attributed to fermentation, the probiotic consortium, or the milk matrix. Further controlled studies are needed to identify the active components and validate the underlying mechanisms.

## Data Availability

The original contributions presented in this study are included in the article/Supplementary Material. Further inquiries can be directed to the corresponding author.

## Supplementary Materials

The following supporting information can be downloaded at: https://www.mdpi.com/article/10.3390/foods15203680/s1, Figure S1: (A) LDA score histogram on LEfSe analysis; (B) Spearman correlation analysis between the unique microbiota in the LPH group and the four lipid parameters; (C) cladogram based on LEfSe analysis. (D) MetagenomeSeq analysis; Figure S2 Spearman correlation analysis between the MC and LPH groups: (A) fecal differential metabolites and four blood lipid parameters; (B) hepatic differential metabolites and four blood lipid parameters; and (C) hepatic differentially expressed genes and four blood lipid parameters. Table S1: Experimental materials; Table S2: Primer sequences; Table S3: Antibody supplier and catalog number; Table S4: Nutritional composition of FMM; Table S5: Significantly altered metabolites common to fecal and liver metabolomic profiles.
