# Supplementary material for: Probiotic-Fermented Mare’s Milk Ameliorates High-Fat Diet-Induced Dyslipidemia Through Coordinated Gut-Liver Metabolic Remodeling
Source: Foods. 2026 Oct 16;15(20):3680. doi: 10.3390/foods15203680 (PMC13703706; doi:10.3390/foods15203680)
Supplement: Supplementary file 1 [file foods-15-03680-s001.zip › foods-4577207-supplementary.pdf]

Supplementary Materials for

Probiotic-Fermented Mare's Milk Ameliorates High-Fat Diet-Induced Dyslipidemia through Coordinated Gut-Liver Metabolic Remodeling

Rui Zhang[a], Chao Yin[a], Naizire Paerhati[a], Jianbao Ma[b], Nayilan Zainiding[a], Sumaierye Batuer[a], Gulibahaer Kawuli[a,*]

[a] *School of Pharmacy, Xinjiang Medical University, Xinjiang, Urumqi 830017, China*

[b] *School of Nursing, Xinjiang Medical University, Xinjiang, Urumqi 830017, China*

* Correspondence author:

Gulibahaer Kawuli: Guli5573@163.com; School of Pharmacy, Xinjiang Medical University, Xinjiang, Urumqi 830017, China

Supplementary Figure S1

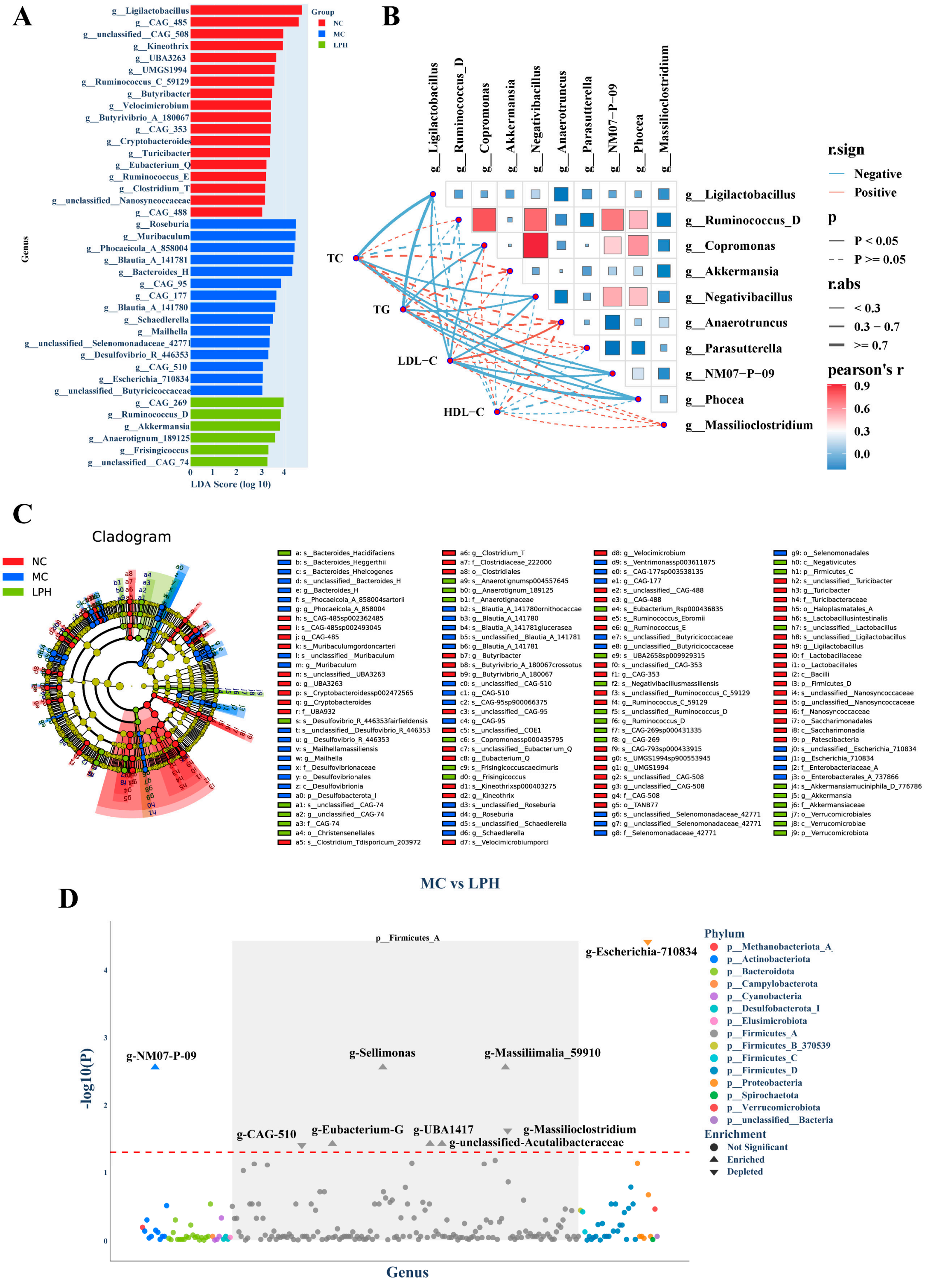

Figure S1. (A) LDA score histogram on LEfSe analysis; (B) Spearman correlation analysis between the unique microbiota in the LPH group and the four lipid parameters; (C) cladogram based on LEfSe analysis. (D) MetagenomeSeq analysis

Supplementary Figure S2

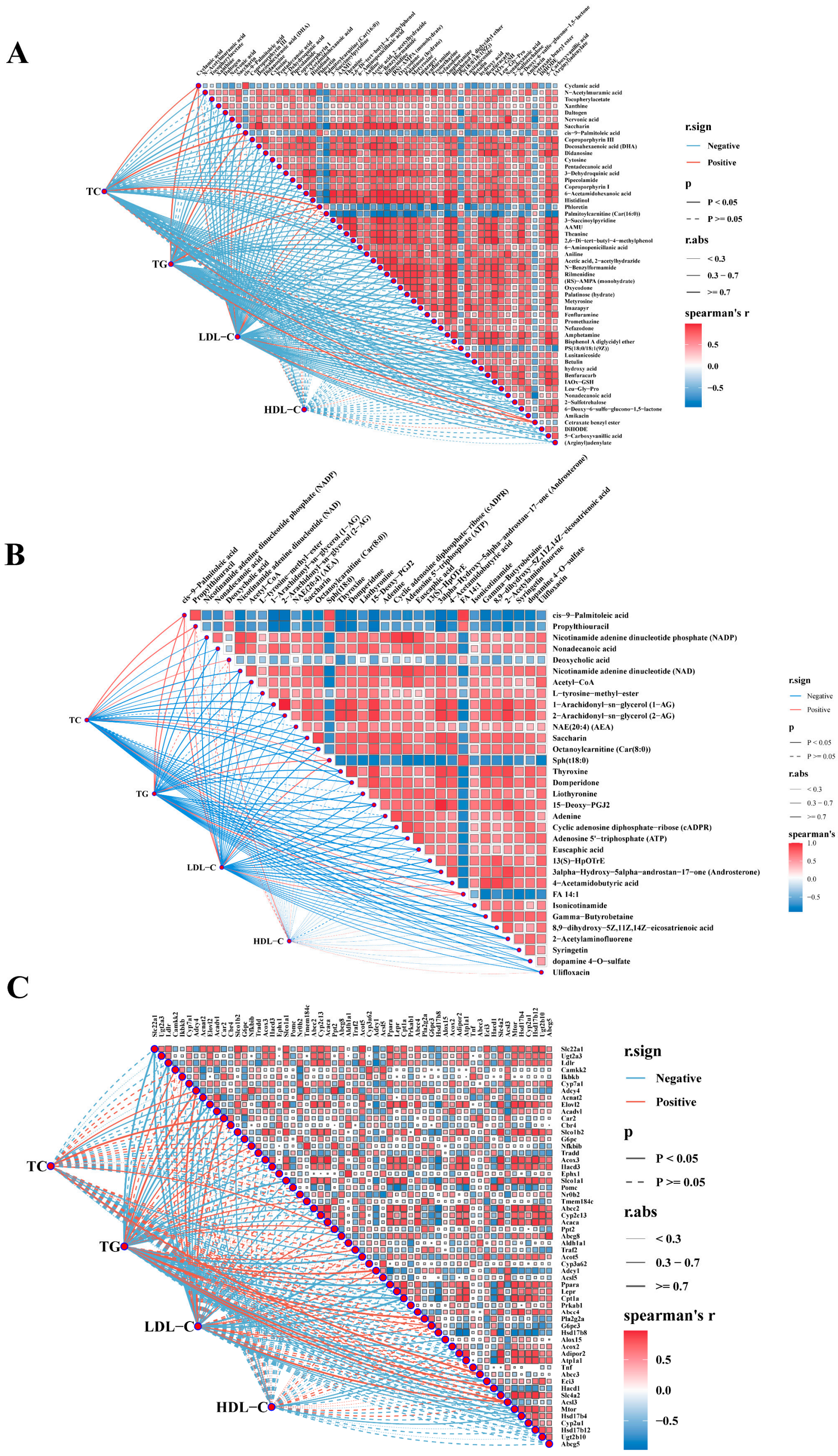

Figure S2. Spearman correlation analysis between the MC and LPH groups: (A) fecal differential metabolites and four blood lipid parameters; (B) hepatic differential metabolites and four blood lipid parameters; and (C) hepatic differentially expressed genes and four blood lipid parameters.

Table S1

Experimental materials

Name	Art. No.	vender
RNA-easy Isolation Reagent	R701-01	Vazyme, Nanjing, China
HiScript III RT Super Mix for qPCR (+gDNA wiper)	R323-01	Vazyme, Nanjing, China
SupRealQ Ultra Hunter SYBR qPCR Master Mix(U+)	Q713-02	Vazyme, Nanjing, China
Total cholesterol assay kit	A111-1-1	Jian Cheng, Nanjing, China
Triglyceride assay kit	A110-1-1	Jian Cheng, Nanjing, China
Low-density lipoprotein cholesterol assay kit	A113-1-1	Jian Cheng, Nanjing, China
High-density lipoprotein cholesterol assay kit	A112-1-1	Jian Cheng, Nanjing, China
Aspartate aminotransferase Assay Kit	C010-2-1	Jian Cheng, Nanjing, China
Alanine aminotransferase Assay Kit	C009-2-1	Jian Cheng, Nanjing, China

Table S2

Primer sequences

GAPDH	F	TTCAGCTCTGGGATGACCTT
	R	TGCCACTCAGAAGACTGTGG
CYP7A1	F	GACTTCACTCTCCATCTTGAGGAC
	R	AAGGGTCTGGGTAGATTTCAGGAT
PPARα	F	GAAGCCTACCTGAAGAAC
	R	CGGATTGTTGCTAGTCTT
ABCG5	F	GCAGGTGGTGATTATGCGTC
	R	CTCTCAGCATGGGAAAGAGGT
ABCG8	F	GATAAACGGGCAACCCAGCA
	R	ACGTCTTCCACCCGTTTGT
CPT1a	F	GGCATAAACGCAGAGCATTCCTG
	R	CAGTGTCCATCCTCTGAGTAGC
MRP2	F	CTGTTCTGGTGTGGATTCCCTTGG
	R	GAATACGCCGCATAAGACCGAGAG

Table S3

Antibody supplier and catalog number

Antibody	Supplier	Catalog No.
CYP7A1	Abcam	Ab65596
PPARα	ABclonal	A25296
CPT1a	ABclonal	A22899
ABCG5	Abmart	TD8401S
ABCG8	Abcam	Ab223056
MRP2	Abcam	Ab203397

Table S4

Nutritional composition of FMM

Nutrient	Per 100 mL	NRV (%)
Energy	210 kJ	3
Protein	1.9 g	3
Fat	1.6 g	3
Carbohydrate	7.0 g	2
Sodium	13 mg	1

Table S5

Significantly altered metabolites common to fecal and liver metabolomic profiles

Name	mz	Formula
cis-9-Palmitoleic acid	253.217	C16H30O2
Myristic acid	227.2013	C14H28O2
LPE (14:0)	424.247	C19H40NO7P
Nonadecanoic acid	297.2795	C19H38O2
PC (37:4)	796.5832	C45H82NO8P
Pentadecanoic acid	241.2171	C15H30O2
Dodecanoic acid	199.1702	C12H24O2
Lumichrome	241.073	C12H10N4O2
12-Methyltridecanoic acid	227.2011	C14H28O2
Hexadecanedioic acid	285.2072	C16H30O4
Cer(d18:1/16:0)	538.5184	C34H67NO3
Saccharin	181.9913	C7H5NO3S
PC (38:8)	802.536	C46H76NO8P
Prolylhydroxyproline	229.118	C10H16N2O4
PC (42:11)	852.5491	C50H78NO8P
3-Aminopiperidine-2,6-dione	129.0658	C5H8N2O2
o-Tyrosine	182.0807	C9H11NO3
1-Lauryl-2-pyrrolidone	254.2475	C16H31NO
2-methoxyethyl 4-(3-hydroxyphenyl)-7-(2-methoxyphenyl)-3-2-methyl-5-oxo-4,6,7,8-tetrahydro-1H-quinoline-3-carboxylate	462.1918	C27H29NO6
Pentylenetetrazole	139.0976	C6H10N4
2-[[(5Z,8Z,11Z,14Z)-icosa-5,8,11,14-tetraenoyl] amino]propanoic acid	374.27	C23H37NO3
(4aR,6aS,6bR,10S,12aS,14bR)-10-hydroxy-2,2,4a,6a,6b,9,9,12a-octamethyl-3,4,5,6,6a,7,8,8a,10,11,12,14b-dodecahydro-1H-picen-13-one	441.3715	C30H48O2
H-Glu-Val-D-Phe-OH	392.183	C19H27N3O6
9-Octadecenoic acid (9Z)- (1R)-1-[[[(2,3-dihydroxypropoxy) hydroxy phosphinyl]oxy]methyl]-2-[(1oxooctadecyl) oxyy]ethyl ester	775.5485	C42H81O10P

2,4-Dichloro-3-ethyl-6-aminophenol	204.0001	C8H9Cl2NO
3-[[4-(4-Oxidanylidene-3h-Quinazolin-2-Yl)phenyl]methyl] imidazolidine-2,4-Dione	333.1016	C18H14N4O3
alpha-amino-1H-pyrazole-1-propanoic acid	154.062	C6H9N3O2
5-chloro-2,4-dihydroxy-6-methyl-3-[2E,4E)-3-methyl-5-(1,2,6 -trimethyl-5-oxocyclohex-3-en-1-yl)penta-2,4-dienyl]benzalde hyde	401.1557	C23H27ClO4
1-Benzyl-3-thiazol-2-yl-thiourea	248.0344	C11H11N3S2
1-(4-Methoxyphenyl) piperazine	193.1312	C11H16N2O
3-Aminopyrrolidine-3-carboxylic acid	131.0812	C5H10N2O2
1-Hydroxy-13-(4-oxoquinazolin-3-yl)-10-propan-2-yl-8,11-dia zatetracyclo [6.6.1.02,7.011,15]pentadeca-2,4,6-triene-9,12-dione	431.1672	C24H22N4O4
1-(4-Chlorophenyl)-3-cyclopentylthiourea	255.0739	C12H15ClN2S
9(10)-EpOME	297.242	C18H32O3
Incensole acetate	349.2729	C22H36O3
H-DL-Glu-DL-His-DL-Pro-NH2	379.1791	C16H24N6O5
Bucladesine	468.1293	C18H24N5O8P
Unii-73hdp58zfk	241.1194	C11H18N2O4
13,17-Dihydroxy-6-(3-hydroxyprop-1-en-2-yl)-16-methoxy-2, 7,20-trioxapentacyclo [11.8.0.03,11.04,8.014,19] henicosa-3(11),4(8),9,14,16,18-hexaen-12-one	411.1085	C22H20O8
Lovastatin Acid	421.2595	C24H38O6
Chenodeoxycholylphenylalanine	540.3655	C33H49NO5
Methyl 3-[3,4-dihydroxy-5-(3-methylbut-2-enyl) phenyl]-2-[4-hydroxy-3-(3-methylbut-2-enyl) methyl]-4-methoxy-5-oxofuran-2-carboxylate	521.2212	C30H34O8
3-Hydroxy-2-[1-[2-[(4-hydroxybenzoyl) amino]-3-methylbutanoyl] pyrrolidine-2-carbonyl] amino] benzoic acid	468.1719	C24H27N3O7
Octadec-9-ene-1,18-dioic-acid	311.2223	C18H32O4
10-(3,4-Dimethyl-5-pentylfuran-2-yl) decanoylcarnitine	480.3669	C28H49NO5
